# Work history, economic resources, and women’s labour market withdrawal after the birth of the first grandchild

**DOI:** 10.1007/s10433-019-00525-x

**Published:** 2019-07-12

**Authors:** Francesca Zanasi, Inge Sieben, Wilfred Uunk

**Affiliations:** 1 Department of Sociology, TS Social and Behavioural Sciences, Tilburg University, Warandelaan 2, 5037 AB Tilburg, The Netherlands; 2 Chair of Sociology I, Otto-Friedrich-Universität Bamberg, Feldkirchenstr. 21, 96052 Bamberg, Germany

**Keywords:** Grandparenthood, Life course, Work history, Economic resources, Labour market withdrawal, English longitudinal study of ageing

## Abstract

Typically, grandmothers are actively involved in the lives of their grandchildren, most frequently as care providers. At the same time, these individuals become grandparents while still employed. These two roles—of active grandparent and worker—might conflict, since both demand time and energy. This study examines whether the birth of the first grandchild leads to labour market withdrawal for women, and whether there are differences between grandmothers according to their work history and household economic resources. We considered the work history of women both as a measure of work–family preferences and a source of opportunities and constraints to labour market behaviour later in life. Our analyses of data from the English Longitudinal Study of Ageing (ELSA) 2002–2017 using hybrid logistic models show that the probability of labour market withdrawal increases after the birth of the first grandchild. Women who had continuous working careers, or short employment interruptions, were more likely to withdraw from the labour market after the birth of the first grandchild than their counterparts with non-continuous careers, as well as women living in wealthy households. The explanation lies in the lower opportunity cost these women encounter in withdrawing from the labour market. Our findings relate to policies aimed at increasing retirement ages all over Europe, advocating that these measures could conflict with grandmothers’ involvement in their grandchildren’s lives.

## Introduction

All over Europe, increased life expectancy makes it common for grandchildren to grow up while their grandparents are still alive, and research on the multifaceted role of grandparents has begun to proliferate (for a literature review, see Hank et al. 2018). Scholars have widely investigated the role of grandparents as childcare providers (Attias-Donfut et al. 2005; Hank and Buber 2009) whose positive externalities extend to at least two generations. On the one hand, grandparental care supports younger generations’ employment (Dimova and Wolff 2011), especially in those institutional settings where formal childcare is rarely or narrowly provided (Bordone et al. 2017). On the other hand, becoming a grandparent is experienced as a highly positive life transition by individuals (Mahne and Motel-Klingebiel 2012). That is, spending time with grandchildren provides emotional gratification and a sense of belonging and usefulness, with positive effects on health and life satisfaction (Arpino and Bordone 2014; Mahne and Huxhold 2015; Di Gessa et al. 2016).

Scientific interest in grandparents has coincided with major policy reforms all over Europe aimed at raising pension ages (OECD 2017). Scholars have warned that keeping older workers in the labour market could conflict with their involvement in grandchildren’s lives (Gray 2005), and that this involvement is related to early retirement preferences (Hochman and Lewin-Epstein 2013). Indeed, midlife individuals are likely to be in employment when they become grandparents, as all over Europe the transition to grandparenthood typically precedes retirement by at least 5 years (Leopold and Skopek 2015). In recent years, several cross-national studies in Europe have confirmed that becoming a grandparent (Van Bavel and De Winter 2013) and providing childcare (De Preter et al. 2013) are associated with early retirement. Single-country studies focusing on the USA, Sweden, and Austria reached similar conclusions (Lumsdaine and Vermeer 2015; Frimmel et al. 2017; Kridahl 2017; Rupert and Zanella 2018).

Our study focuses on England, where the work–grandchild conflict and its consequences for labour market participation have received relatively little attention, especially regarding women, who bear the burden of care responsibilities and are the most likely to provide grandchild care (Gray 2005). The English case is of interest for several reasons. Firstly, in England, grandparents, especially grandmothers, have a complementary role to formal childcare services (Wheelock and Jones 2002; Gray 2005). In England, childcare services are market provided, with state-funded places allocated through means-testing (Lewis and West 2017). The cost of childcare is among the highest in OECD countries, amounting to 26.6% of family income (OECD 2011). To increase maternal employment the state employs tax credits and subsidizes free hours of childcare for low-income families. Nevertheless, these measures do not fully account for families’ childcare needs, especially for those families working non-standard hours. Moreover, they have been the target of recent austerity measures (Lewis and West 2017). Thus, over a third of English families rely on informal care, mainly provided by grandparents, against 20% of families in France and 0.1% in Denmark (OECD 2011). Secondly, English grandparents are expected to be economically active: early retirement solutions are not easily provided (Schils 2008) and the statutory pension age for women is rising rapidly from 60 to 65, bringing it in line with the pension age for men. Finally, England has a contribution-based pension system (Schils 2008). Contributions to the basic state pension are acquired via years of employment, and individuals can opt into voluntary private pensions to supplement the basic state pension (Gardiner et al. 2015). As women’s reproductive labour is often linked with discontinuous working careers, they have a limited opportunity to build up state, private, or occupational pensions, with consequences for their pension incomes in later life (Ginn and Arber 1996; Evandrou and Glaser 2003; Sefton et al. 2011; Gardiner et al. 2015) and retirement timing (Finch 2014). Thus, women’s life courses and economic resources are crucial factors in both the attraction and feasibility of labour market withdrawal (LMW) after the birth of a grandchild.

## Study aim and hypotheses

In this study, we focused on the relation between the birth of the first grandchild and midlife women’s working careers, and specifically on the probability of their LMW. Moreover, we add to the existing literature by considering differences between grandmothers in terms of work histories and economic household situations.

When studying working histories, we adopted a life course approach. Deciding how to reconcile grandchild care provision with paid work depends on women’s previous work–family history. Work–family histories could signal, on the one hand, persisting preferences for family or work (in a perspective we call “attachment hypothesis”), but, on the other hand, they could determine the economic affordability of LMW (in a perspective we call “opportunity cost”) when women become grandmothers. When looking at household’s economic situations, we adopted a couple perspective, considering that labour market decisions are taken in a family context. Underpinned by the theoretical background below, three hypotheses guided our analyses:

- Compared to women with discontinuous working careers, women with continuous working careers are *less* likely to withdraw from the labour market after the birth of the first grandchild, due to persisting preferences for work over family.
- Compared to women with discontinuous working careers, women with continuous working careers are *more* likely to withdraw from the labour market after the birth of the first grandchild, due to accumulation of pension contributions and economic independence.
- Compared to women from low-income households, women from high-income households are more likely to withdraw from the labour market after the birth of the first grandchild.

Grandmothers will already have had to make significant decisions about work and care at least once in their adult lives, around the birth of their own child(ren). They will have decided which strategies to implement in order to reconcile their work and family lives, such as delayed labour market entry, LMW, part-time working, or prolonged work. The adopted work–family strategy has distinct implications for the study of grandmothers’ work decisions (Pienta et al. 1994; Hank 2004; Finch 2014).

On the one hand, we may assume that the priority given to work/family throughout one’s working career is an indicator of work/family orientations. Research shows that women who continued to work during their childbearing period were more likely to be at work thereafter (Pienta et al. 1994; Hank 2004; Finch 2014). The same holds true for women postponing childbearing (Pienta 1999; Stafford et al. 2018). These studies used the so-called “attachment hypothesis” to explain the underlying mechanism. That is, in cases where women have invested in their personal attainment and human capital accumulation, they hold stronger ties to the labour market, leading to later retirement ages (Pienta 1999; Hank 2004). Hence, grandmothers may reproduce preferences and practices already put in place when they became mothers; those who have had a continuous working career could be *less* likely to withdraw from the labour market in the late stage of their career when they have grandchildren than women who had a discontinuous working career due to care responsibilities (Hypothesis 1a).

On the other hand, the decisions about paid work taken earlier in life, for example, around childbirth, contribute to economic independence and the accumulation of pension wealth; the years spent working have long-term consequences in terms of retirement eligibility and the economic affordability of LMW. Finch (2014) discussed the opportunity cost of retiring for those women who have had career breaks, usually experienced by women for care responsibilities, due to the resulting low levels of pension wealth. To receive the full state pension in England, individuals must either meet the state pension age or have paid a certain amount of National Insurance (NI) contributions.^1^ Individuals contributing for a lesser number of years receive a lower amount. Some workers have the option to maintain a private pension scheme, but this is rarely the case for women (Gardiner et al. 2015), which means that they are more often forced to rely on the flat-rate state pension alone. Additionally, the likelihood of receiving income from a private pension fund, and the amount received, is closely related to the individual’s employment pattern (Ginn and Arber 1996). Therefore, women who have had a continuous working career might be *more* likely to withdraw from the labour market when they have a grandchild compared to women who have had a discontinuous working career (Hypothesis 1b), because LMW is feasible both economically and from the perspective of pension eligibility criteria.

When investigating the late-life career decisions of women, it is important to consider the role played by *current* economic resources at the household level. It is an additional way to investigate the opportunity cost of LMW for women, because it includes all resources available in the household. A large body of literature shows that, in many European countries, a husband’s elevated occupational position is related to a reduction in a wife’s work commitment, mainly due to an increased specialization of tasks between the spouses (Blossfeld and Drobnič 2001). So, even women who did not themselves accumulate economic resources and pension wealth might consider LMW as a viable option when they are part of a high-income household. Total family income can thus make up for a lack of economic independence or an inability to meet the eligibility criteria for retirement. Thus, women who are part of high-income households could be more likely to withdraw from the labour market when they have grandchildren than their economically less advantaged counterparts (Hypothesis 2).

## Material and method

### Data

We employed the first eight waves of the English Longitudinal Study of Ageing (ELSA) 2002–2017, which is a biannual panel study on health, economic position, and quality of life among individuals older than 50 and their partners, living in private households (Marmot et al. 2018).^2^ We selected women between 50 and 65 years old and excluded those who had never done paid work and/or were childless. Furthermore, only those respondents who participated in wave 3 (containing information on previous life course) were included in the sample. After these restrictions and excluding observations with missing values in the variables of interest, the final sample comprised 2366 women and 10,207 person–wave observations (average 4.4 observations per individual).

### Variables

The dependent variable was dichotomous, capturing whether the individual was not in paid work and based on self-report to be economically inactive (looking after home/family) or formally retired.

The main independent variable was the birth of the first grandchild. In fact, research shows that first-born or only children more often receive grandparental childcare than second or subsequent children (Fergusson et al. 2008). The respondents were asked to report on the number of grandchildren they had, and from this we created a dummy variable that was equal to (1) if the number of grandchildren changed from 0 to 1 between two waves. This strategy was successfully used by other scholars (e.g. Lumsdaine and Vermeer 2015), since information on the date of birth of the oldest grandchild was not available.^3^

The moderating variables capturing women’s work history and economic resources did not vary across waves. To operationalize women’s work history, we performed sequence analysis (see “Method" and "Analytical approach” sections below) on retrospective information collected in wave 3. This led to the identification of four groups of women according to their work history between ages 18 and 45: (1) women who had largely continuous working careers, with a maximum 1 year not in paid work, e.g. on maternity leave; (2) women who had short (1–5 years) employment interruptions for family-related reasons; (3) women who had long (6–27 years) employment interruptions for family-related reasons; and (4) a residual category of women with employment interruptions for other reasons.

Current economic resources were measured by the yearly income of the household at the baseline. In this way, we made sure that this moderating variable was not sensitive to moves in/outside the labour market. The measure was adjusted against the Retail Price Index (RPI) of 2015. The variable was at the couple level and included individual and spouse earnings, family capital income (self-employment earnings, rental income from property, interest income from financial assets), individual and spouse private pensions or annuities from employers, individual and spouse incomes from public pensions (old age, disability), and other government transfers (veteran benefits, welfare benefits, worker’s compensation benefits, unemployment benefits). We divided respondents into three groups, identified by income terciles calculated on the income distribution of the year the individual was observed for the first time in the sample.^4^

We included a set of control variables. Among time-varying variables, we included age (in categories), which is strongly related to the transition to both grandparenthood and retirement, and partner’s work status (not married, partner not employed, partner employed, partner of other status) as spouses tend to synchronize retirement (Henretta et al. 1993), and having a partner is related to higher family income (Finch 2014). As an enabling factor for grandparental care (Gray 2005; Hank and Buber 2009), we measured the proximity of residence with a dummy variable indicating whether the woman had weekly contact with any of her children in person, and subjective health status (good, fair, bad). Finally, we included life course time-constant characteristics: educational level (less than college, some college, or another kind of qualification), as an additional indication of women’s labour market attachment; birth cohort (before/after 1950), capturing different retirement regulations; number of children, because the greater the number of children, the greater the (eventual) work interruptions as well as the adult children’s need of support; and age at motherhood, because it is related to the timing of retirement and grandparenthood.

### Analytical approach

As mentioned in “Variables” section, we performed sequence analysis to operationalize the work history variable, relying on retrospective information from wave 3. Respondents were asked the start and end date of each of their employment spells, as well as their status between them. From this information, each year in the life of each respondent was assigned to a certain state, namely persistence in school, gap between school and work (i.e. delayed entry in employment), employment, economic inactivity for family-related reasons (including maternity leave), and a final category with other states (e.g. prison, disability, unemployment, travelling). After the identification of the individual life sequences, Optimal Marching Analysis (OMA) was used to compute a matrix of dissimilarities between pairs of sequences that served as input for cluster analysis (Abbott and Tsay 2000). The costs of substitution set to build the matrix were based on the transition probabilities between statuses empirically observed in the data.

The clustering procedure (with Ward’s algorithm) provided standard goodness of fit statistics (Calinski Harabasz pseudo-F statistics and Duda Hart pseudo-T-squared), which made us decide on a four-cluster solution: women (1) with continuous careers; (2) with short employment interruptions (regardless of the reason of interruption); (3) with long employment interruptions for family-related reasons; and (4) with long employment interruptions for other reasons. We slightly altered the clusters to have a theoretically informed categorization of women. In particular, we aimed at clearly distinguishing women’s work histories on the basis of the reason for employment interruptions. Firstly, in cluster (1) we retained only women who had employment interruptions shorter than or equal to one year, for example maternity leave or short unemployment spells. Secondly, the cut-off point between short and long employment interruptions was set to 5 years, which is the age children begin compulsory education and are less in need of childcare. Thirdly, in cluster (2) we included only women with short employment interruptions for family-related reasons. Finally, concerning women in cluster (2) and cluster (3) who experienced *both* short employment interruptions for family-related reasons *and* for other reasons, we included only women who *also* had up to 2 years of employment interruptions for other reasons, and moved women with other kinds of career trajectories into cluster (4).

To investigate the relation between the transition into grandparenthood and the transition out of the labour market, we used between–within random effects logistic models, also called hybrid models (Allison 2009; Schunck 2013). This analytical strategy offers the advantages of fixed-effects models, allowing the decomposition of the *between*- and *within*- individual effects for time-varying covariates. At the same time, it has a more flexible setup, also estimating the coefficients for variables that do not vary within individuals, such as the variables work history and total family income. In this study, we decomposed the time-varying predictor “first grandchild born” into two parts: the individual’s mean value over time (*between*-individual component) and the deviation from this person-specific mean (*within*-individual component). The *within*-individual component was based on changes over time and resembled estimates of individual fixed-effects models. The score compared the outcome before/after a change in predictor, based on observations belonging to the same individual. In our case, it showed the difference in log-odds of LMW before and after having a grandchild for the same person, namely how *becoming* a grandmother was associated with LMW. In addition, the *between*-individual component accounted for all unobserved time-constant individual characteristics. In other words, it captured all those unobserved variables correlated with grandparenthood that were also correlated with LMW. The score was based on the comparison between women who were already grandmothers and women who were not, namely whether grandmothers, when compared to non-grandmothers, were more likely to withdraw from the labour market.

Since in logistic regression models it is problematic to interpret log-odds ratios (Mood 2010), for each model we presented the results in terms of average marginal effects (AMEs), namely the average differences in probability of LMW between the categories of the variables of interest. We set the statistical significance level at *p* < 0.05. The statistical analyses were carried out with Stata 14 software (StataCorp 2015).

## Results

Table 1 shows the characteristics of the sample. A good third of the women (33%) were already outside the labour force (of which 17% were retired and 16% looking after home or family) at the beginning of the observation window, and just over half (53%) of them were already grandmothers when they entered the survey. As to events occurring within the observation window, 24% of the women became grandmothers for the first time, and 43% recorded at least one transition out of paid employment.

**Table 1:** Characteristics of the sample, *n* (individuals) = 2366 and *N* (observations) = 10,207

Variable	Category	At survey entry	%	*N*
Work status	Not in labour force	797	33	4257
	Other statuses	1569	67	5950
	Women who withdrew from LM during observation period	1008	43	
Grandchild	No grandchild born	1117	47	3795
	First grandchild born	1249	53	6412
	Women who became grandmother during observation period	563	24	
Work history	Continuous	488	21	2157
	Short interruptions	456	19	2015
	Long interruptions	994	42	4290
	Other	428	18	1745
Total family income	1st tercile	790	33	3129
	2nd tercile	790	34	3376
	3rd tercile	786	33	3702
Age	50/55	1401	59	2818
	56/60	529	22	3611
	61/65	436	19	3778
Educational level	Less than college	1368	58	5805
	Some college	816	34	3662
	Else	182	8	740
Partner’s work status	No partner	572	24	2441
	Partner employed	1059	45	4006
	Partner not employed	451	19	2645
	Partner other status	284	12	1115
Birth cohort	Before 1950	1376	58	5130
	After 1950	990	42	5077
Weekly contact with children	No	529	22	2902
	Yes	1837	78	7305
Subjective health (good/bad)	Mean (sd)	0.26 (0.54)		
Number of children	Mean (sd)	2.46 (1.25)		
Age at motherhood	Mean (sd)	24.2 (5.13)		
Total observations				10,207
Total unique women		2366		

In Table 2, we report the results of between–within random effects logistic analyses. Model 1 shows how the birth of the first grandchild related to LMW when all control variables were added. The coefficient based on the *within*-individual component (0.77) was statistically significant, while the *between*-individual component was not.^5^ This means that *becoming* a grandmother was positively related to LMW, but *being* a grandmother was not. The AME to be outside the labour market *within* women, namely before and after the first grandchild was born, was around 8 percentage points.

**Table 2:** Hybrid models for the probability of LMW, *n* (individuals) = 2366 and *N* (observations) = 10,207

Variable	Model 1			Model 2			Model 3		
	Coeff.		SE	Coeff.		SE	Coeff.		SE
First grandchild born									
Grandchild—within (W)	0.770	***	0.163	1.431	***	0.384	0.114		0.311
Grandchild—between (B)	0.251		0.215	0.960	*	0.402	0.192		0.341
Work history									
Continuous career (reference)									
Short interruptions	0.275		0.226	0.666		0.410	0.273		0.226
Long interruptions	1.228	***	0.196	1.750	***	0.357	1.229	***	0.197
Other	2.195	***	0.239	2.995	***	0.412	2.188	***	0.239
Total family income									
1st tercile (reference)									
2nd tercile	− 0.621	***	0.182	− 0.640	***	0.183	− 0.783	*	0.369
3rd tercile	− 0.685	***	0.196	− 0.710	***	0.197	− 0.697	*	0.344
Interaction terms									
Grandchild (W) × short interruptions				0.142		0.543			
Grandchild (W) × long interruptions				− 0.967	*	0.433			
Grandchild (W) × other				− 1.327	*	0.539			
Grandchild (B) × short interruptions				− 0.649		0.539			
Grandchild (B) × long interruptions				− 0.798		0.465			
Grandchild (B) × other				− 1.272	*	0.541			
Grandchild (W) × 2nd tercile							0.380		0.399
Grandchild (W) × 3rd tercile							1.233	**	0.391
Grandchild (B) × 2nd tercile							0.226		0.444
Grandchild (B) × 3rd tercile							− 0.045		0.428
Age									
50/55 (reference)									
56/60	0.778	***	0.104	0.790	***	0.105	0.777	***	0.105
61/65	3.403	***	0.136	3.408	***	0.136	3.405	***	0.136
Educational level									
(less than) high school									
Some college	− 0.196		0.163	− 0.195		0.163	− 0.195		0.163
Else	− 0.628	*	0.270	− 0.630	*	0.271	− 0.631	*	0.271
Partner’s work status									
No partner (reference)									
Partner employed	− 0.042		0.166	− 0.036		0.166	− 0.046		0.166
Partner not employed	1.785	***	0.167	1.791	***	0.167	1.771	***	0.167
Partner other status	0.830	***	0.184	0.842	***	0.185	0.815	***	0.185
Birth cohort (born after 1950)	− 1.303	***	0.159	− 1.297	***	0.159	− 1.308	***	0.159
Weekly contact with children (yes)	− 0.106		0.102	− 0.105		0.102	− 0.116		0.102
Subjective health (bad)	− 0.052		0.087	− 0.044		0.087	− 0.056		0.087
Number of children	0.033		0.073	0.044		0.073	0.033		0.073
Age at motherhood	0.020		0.018	0.024		0.018	0.020		0.018
Constant	− 2.865	***	0.391	− 3.372	***	0.455	− 2.797	***	0.435
Log-likelihood	− 4358.94			− 4349.73			− 4352.93		

***p < 0.001, **p < 0.01, *p < 0.05

Model 2 adds the interaction term between the grandchild variables (both *within* and *between* components) and women’s work history. We found a statistically significant relation between the transition to grandparenthood (*within* component) and LMW for women who had long employment interruptions for family-related reasons or employment interruptions for other reasons, compared to women with continuous careers, the reference category. No statistically significant difference was found between women who had short employment interruptions and women with continuous working careers. Similarly, Model 3 estimated the interaction effect of total family income and the first grandchild’s birth. The interaction term showed a statistically significant, positive relation between the transition to grandparenthood (*within* component) and LMW for women who belonged to the third tercile of the income distribution compared to women from the first income tercile, the reference category. To the contrary, there was not a statistically significant difference between women belonging to the second and the first income tercile.

**Fig. 1:**
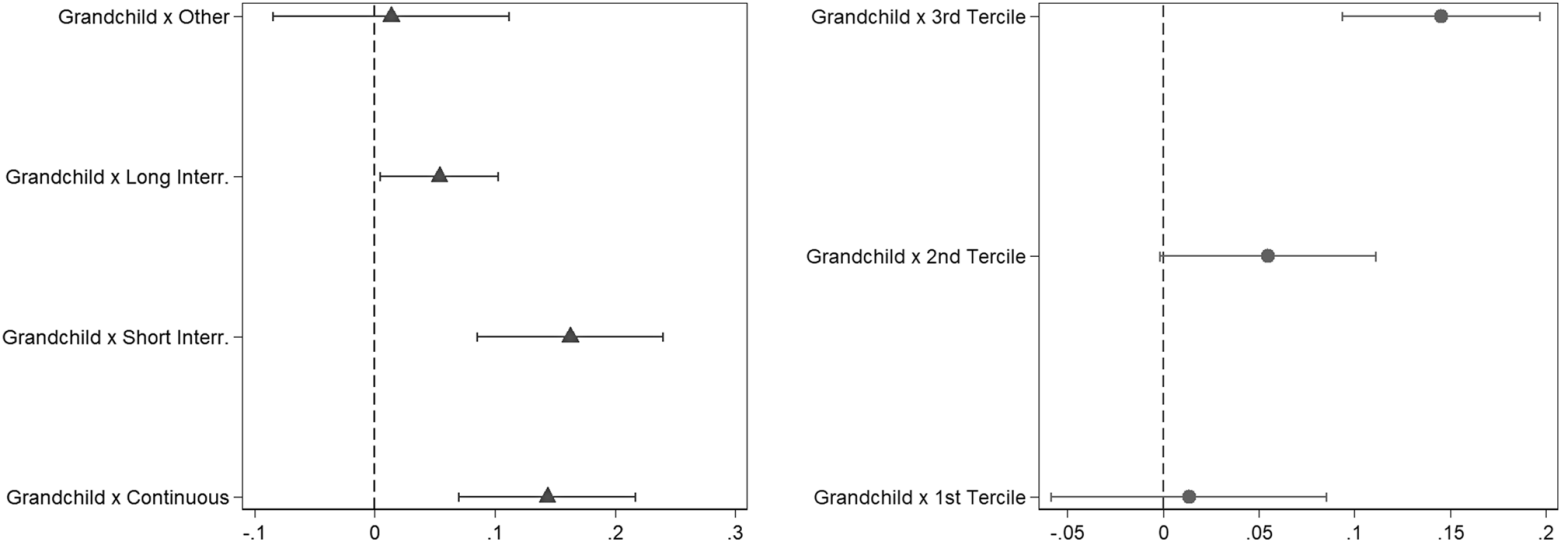
Difference in probability of withdrawing from the labour market before/after the birth of a grandchild, by work history (left panel, triangles) and total family income (right panel, dots). 95% Confidence intervals. *n* (individuals) = 2366 and *N* (observations) = 10,207

Figure 1 displays the AMEs for the probability of being outside the labour market *within* women after the first grandchild’s birth, according to work history (from Model 2, left panel in Fig. 1) and total family income (from Model 3, right panel in Fig. 1). Having continuous careers or careers with short interruptions increased the probability of LMW for women after their first grandchild’s birth by around 15 percentage points. On the other hand, having long employment interruptions for family-related reasons only slightly increased the probability of LMW, and no increase was discernible for women with careers interrupted for other reasons. Turning to total family income, women living in households belonging to the third income tercile were roughly 14 percentage points more likely to withdraw from the labour market after the first grandchild was born, while there was no statistically significant relation between becoming a grandmother and LMW for women belonging to the first and second income terciles.

## Discussion

In this study, we investigated how the birth of the first grandchild relates to grandmothers’ LMW in England by employing recent panel data from the ELSA survey (2002–2017). Our main contribution was the adoption of a life course perspective, central in the research on ageing (Bengtson et al. 2005), because it implies the understanding of late-life events as resulting from the interaction between work history and present contingencies, in our case the birth of the first grandchild. Moreover, we accounted for the fact that labour market decisions are not made in a vacuum, but in a family context (De Preter et al. 2015). That is, current economic resources at the family level could moderate the association between the birth of the first grandchild and LMW. Our results show that the birth of the first grandchild increases the probability of LMW, but differences exist according to grandmothers’ characteristics.

We found confirmation of the “opportunity cost” perspective, namely the idea that privileged women, both in terms of their own working careers and their household resources, are those who can most easily afford LMW upon the arrival of a grandchild. Firstly, the birth of the first grandchild increases the probability of LMW for women with continuous working careers, or with short employment interruptions, confirming our Hypothesis 1b instead of Hypothesis 1a (the “attachment hypothesis”). The amount of time spent not working in these cases has been short enough to avoid resulting in disadvantages in later life, in terms of pension wealth, and they have been able to withdraw to a larger extent when becoming grandmothers. However, this result should be interpreted with caution, because the 95% confidence intervals partially overlap.

Secondly, the birth of the first grandchild raises the probability of LMW for women belonging to high-income families, which is not the case for women from low-income households, confirming our Hypothesis 2. This result is in line with previous studies showing that grandparents providing child care are usually more wealthy (Wheelock and Jones 2002; Gray 2005; Glaser et al. 2013). For this category of women, choosing to withdraw from the labour market after the birth of a grandchild does not result in a high opportunity cost, even if it could lead, for example, to a reduced state pension income. It could be that living with a wealthy partner makes up for eventual foregone earnings caused by LMW. In fact, individual labour market behaviour involves the family as a unit, especially when it comes to women, whose labour market trajectories are strongly intertwined with family responsibilities and the husband’s resources (Henretta et al. 1993; Blossfeld and Drobnič 2001).

This study presents some limitations that ought to be addressed. Firstly, the dependent variable captured self-reported employment status, regardless of the number of hours worked. We are aware of the fact that women could decide to reduce their working hours, instead of dropping out of work altogether, after the grandchild’s birth (see, for example, Rupert and Zanella 2018). Moreover, we did not distinguish between full-time and part-time work with regard to our main moderating variable tapping women’s working history. Our choice is justified by ongoing pension reforms raising pension age (OECD 2017) and thus the urge to understand whether family dynamics could conflict with extended working lives. Surely, this is an interesting and relevant direction for future research. Adjustments in terms of working hours for midlife women could be investigated in the light of (eventual) transitions to part-time work experienced around motherhood. This would further refine the life course approach and the operationalization of the “attachment hypothesis” and “opportunity costs” perspective. Secondly, we were unable to include more detailed measures of the life history of the respondents, such as the kinds of jobs they held. This information, not present in the data, could provide additional insights into the socioeconomic positions of women, further disentangling the constraints and opportunities surrounding LMW. We suggest this as an additional direction for further research, to better understand how inequalities during the life course impact later life, especially around the birth of a grandchild. Finally, it could be argued that adult children adjust their fertility intentions on grandparental availability, in the sense that the grandchild’s birth occurs once the grandparents are retired. This is the case for Italy (Battistin et al. 2014) and for second-order births in the Netherlands (Thomese and Liefbroer 2013). Similar evidence is lacking for England, but studies have pointed out that grandparenthood precedes British women’s LMW by 7 years (Leopold and Skopek 2015). Hence, we believe our study to be well grounded in the field of the consequences of work–family conflict. Further investigation of this reverse relationship, namely how LMW affects the transition to grandparenthood, could shed light on the multiple consequences of rising pension age in terms of fertility.

This article adds England to the collection of single-country studies showing the relationship between the birth of grandchildren and labour market adjustments, which include Austria (Frimmel et al. 2017), Sweden (Kridahl 2017), and the USA (Lumsdaine and Vermeer 2015; Rupert and Zanella 2018). The overlap between grandparenthood and employment is conflictual for midlife individuals living in highly heterogeneous welfare settings, who share the desire to early retire to spend more time with their grandchildren (Hochman and Lewin-Epstein 2013). Thus, the conclusions of the present study go beyond the English context and resonate with several voices advocating for caution in raising the retirement age (see, for example, Glaser et al. 2013). In settings that lack a universal provision of childcare services, keeping older workers in the labour market could lead, over time, to childcare gaps for working parents (Gray 2005; Glaser et al. 2013). Moreover, our study suggests that pension reforms might be effective only in keeping economically worse off grandmothers on the labour market, while better-off women are able to afford retirement or economic inactivity. Low-income families might find simultaneous difficulties in relying on market-provided childcare services and on their older mothers, who are unable to give up their work commitments. In countries such as Sweden, where formal childcare services are universally provided, and grandparental childcare is not driven by need (Igel and Szydlik 2011), grandparents still give up their work commitment (Kridahl 2017). Thus, policies aimed at increasing the labour market participation of older workers are not guaranteed to be effective and may not mitigate financial losses for those aiming to enact the grandparental role. In conclusion, grandmothers should not be overlooked in family policy-making, to ensure that involvement in grandchildren’s lives is not the privilege of a few, and to avoid negative effects on labour market participation and pension wealth.

## Footnotes

1 The flat-rate pension consisting of £122.30 (around €104) per week applies to individuals who reach the state pension age before the 6 April 2016. Regarding pension contributions, individuals born after April 1950 need a total of 30 qualifying years of NI contributions; women born before that date need 39 years of employment to receive a full state pension.

2 Ethical approval for all the ELSA waves is granted by the NHS Research Ethics Committees under the National Research and Ethics Service (NRES).

3 Information about the age of the oldest grandchild was available in the last wave released (8) of the ELSA survey. For those individuals’ in analytical sample who also participated in wave 8 (*n* = 1753), we used retrospective information about the date of birth of the first grandchild and date of retirement. Results point the same way.

4 The cut-off points for each yearly family income distribution were (in Euros): 23,087 and 39,834 for 2002/2003, 36,778 and 53,786 for 2004/2005, 27,006 and 49,355 for 2006/2007, 34,050 and 52,849 for 2008/2009, 33,219 and 51,625 for 2010/2011, 31,199 and 51,322 for 2012/2013.

5 The Wald test of equality of coefficients (based on Chi-square test) shows that the *within* and *between* estimates are different (*p* < 0.05).
